# Extreme REM Rebound during Continuous Positive Airway Pressure Titration for Obstructive Sleep Apnea in a Depressed Patient

**DOI:** 10.1155/2014/292181

**Published:** 2014-05-04

**Authors:** Anna Lo Bue, Adriana Salvaggio, Giuseppe Insalaco, Oreste Marrone

**Affiliations:** Institute of Biomedicine and Molecular Immunology, National Research Council, Via Ugo La Malfa 153, 90146 Palermo, Italy

## Abstract

A 20% increase in REM sleep duration has been proposed as a threshold to identify REM rebound in patients with obstructive sleep apnea (OSA) who start continuous positive airway pressure (CPAP) treatment. We describe the case of one patient with OSA who showed an unexpectedly high degree of REM rebound during titration of CPAP. A 34-year-old man was diagnosed with OSA. He remained untreated for many years, during which he developed systemic hypertension, depression, and severe daytime somnolence. When he was reevaluated sixteen years later, his Epworth sleepiness score was 18, and his OSA had greatly worsened (apnea/hypopnea index: 47, lowest nocturnal saturation: 57%). He underwent a successful CPAP titration during nocturnal polysomnography. Electroencephalographic analysis of the sleep recording revealed a huge amount of REM sleep, accounting for 72% of the total sleep time. When asked, the patient referred that he had suddenly interrupted paroxetine assumption three days before the polysomnography. The very large REM rebound observed in this patient could be due to additional effects of initiation of CPAP therapy and suspension of antidepressive treatment. This case does not report any dangerous consequence, but sudden antidepressive withdrawal could be dangerous for patients with OSA who develop hypoventilation during REM sleep with CPAP application.

## 1. Introduction

Obstructive sleep apnea (OSA) is a common sleep disorder characterized by intermittent partial or complete upper airway obstruction during sleep. It is associated with nocturnal intermittent hypoxemia and with an altered sleep structure that is characterized by a sleep fragmentation, an increase in light NREM sleep, a decrease in REM sleep, and an even more marked decrease in slow wave sleep [1]. As the termination of most respiratory events is associated with an arousal, subjects with severe OSA usually show the worst sleep disruption.

Continuous positive airway pressure (CPAP) readily and fully prevents obstructive respiratory events once it is applied at an appropriate level and is the gold standard treatment for OSA. As respiratory events are eliminated, marked increases in either slow wave sleep and/or REM sleep [2, 3], an increase in REM density [3], and a strong decline in arousal frequency [4, 5] may occur. Amounts of slow wave and REM sleep achieved during the first night of CPAP application may exceed those usually observed in normal subjects. The rebound in these sleep stages tends to decrease progressively in the following nights of treatment [2], and, within one month from CPAP initiation, the rebound in REM sleep has already come to an end [6]. Improvement in sleep structure is associated with a subjective feeling of improvement in sleep quality [7] and is believed to be one cause of the decrease in sleepiness that is usually observed with OSA treatment.

The amount of rebound of REM sleep during the first night of CPAP application varies among patients [5, 8]. According to a recent paper, a ≥20% increase in REM duration in the total sleep time should define a REM rebound [8]. In this report, we describe the case of a subject in whom the simultaneous effects of CPAP application and antidepressive drug withdrawal were followed by a REM sleep duration longer than has ever been described, to our knowledge, in any patient with OSA.

## 2. Case Presentation

A 34-year-old man was referred to our sleep laboratory due to symptoms of heavy snoring that worsened in the supine position, nonrestorative sleep, and tendency to fall asleep in nondemanding situations since about 5 years. Habitual sleep duration was 5 to 6 hours per night. The patient did not refer to any past or current illness that is worth mentioning, did not take any medication on a regular basis, and did not complain of any symptom in addition to the sleep disturbances. He was a heavy smoker (about 30 cigarettes/day), but his spirometry was normal (FEV1/FVC:  78% and FEV1:  88% of predicted).  He showed a moderate weight excess (BMI: 27.1 kg/m^2^) and a moderately deviated nasal septum. His office blood pressure was normal (120/85 mmHg). Physical examination was unremarkable.

A polysomnographic exam was performed. Numerous obstructive apneas and hypopneas were observed in the supine posture. The number of apnea and hypopnea events per hour of sleep time (apnea/hypopnea index [AHI]) was 15.7, with a remarkable posture effect (AHI supine: 42.6 and nonsupine: 1.4). REM sleep was totally spent in lateral posture and was not associated with any respiratory disorder. The lowest oxyhemoglobin saturation (SaO_2_) was 85%, and the time spent with SaO_2_ < 90% was 3.3%. A moderate OSA was diagnosed. A diet, nasal surgery, and positional therapy were recommended.

The patient came back sixteen years later. He had undergone nasal surgery twelve years before, but his weight had increased (BMI: 30.4 kg/m^2^). Ten years before, he had become hypertensive, and he was currently treated with carvedilol, telmisartan, and hydrochlorothiazide. Besides, he had shown depressive symptoms and had begun treatment with paroxetine five months before, at a dosage of 20 mg/day. He had not modified his smoking habits. His current spirometry values were FEV1/FEV:  75% and FEV1:  68% of predicted. He complained of a severe worsening of daytime sleepiness and sleep quality and referred to a very fragmented and restless sleep. He denied cataplexy, sleep paralysis, or hallucinations. Subjective sleepiness evaluated with the Epworth sleepiness scale gave a score of 18.

A nocturnal cardiorespiratory monitoring was performed. Frequent obstructive apneas, mixed apneas, and hypopneas were observed irrespective of posture. The AHI was 47.1. The lowest SaO_2_ was 57%, and the time spent with SaO_2_ < 90% was 31.9%.

Treatment with CPAP was recommended and the patient was scheduled for a nocturnal polysomnography for CPAP titration.

**Figure 1:**
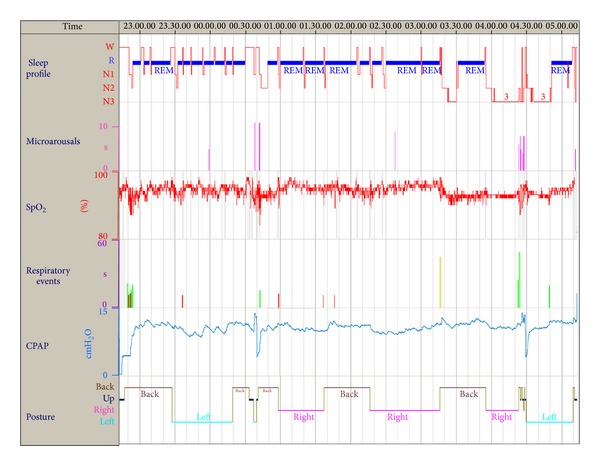
Analysis of the nocturnal polysomnography during automatic CPAP titration. From top to bottom: hypnogram, microarousals, oxyhemoglobin saturation, respiratory events (brown: flow limitations, green: hypopneas, and red: central apneas), CPAP level, and body posture.

During the polysomnography, an automated CPAP was applied (ResMed, AutoSet T, Sydney, Australia). The patient readily fell asleep (sleep latency to stage N1: 6 minutes) and showed a very short REM latency (3 minutes). Then, most of his sleep was represented by REM (71.6% of the total sleep time), while only few arousals and awakenings occurred (arousal index: 1.7 and awakening index: 4.3). Mean CPAP administered during the night was 11 cm H_2_O; 95% of night time was spent with CPAP levels <12.4 cm H_2_O. CPAP application satisfactorily corrected respiratory disorders during both NREM and REM sleep (AHI: 1.9; lowest SaO_2_: 84%, mean SaO_2_ during effective CPAP: 94%) (Figure 1). A fixed CPAP pressure of 12 cm SaO_2_ was considered adequate for treatment.

The following day, when asked, the patient referred that he had suddenly interrupted paroxetine assumption 3 days before. Fixed CPAP at 12 cm H_2_O was prescribed. A new polysomnography was planned for the next month to explore if chronic CPAP treatment and prolonged withdrawal of paroxetine assumption could revert sleep structure to normal. Four months later the patient came back. At the clinical control, the patient had no residual daytime sleepiness; then MSLT was not performed. He had regularly used CPAP (mean use according to the machine time counter: 5.3 hours/night) but referred that he had to resume paroxetine assumption. At the polysomnographic study with fixed CPAP at 12 cm H_2_O, he showed a decrease in REM duration to 57.5 minutes (15.7% of total sleep time) with a REM latency of 38.5 minutes. AHI was 0.2 and lowest SaO_2_ was 89%.

## 3. Discussion

Mild to moderate OSA often shows a progressive increase in severity if left untreated even without any augmentation in body weight, while it may markedly worsen if weight increases [9, 10]. After initial diagnosis, the patient of this study was not effectively treated. In fact, nasal surgery, if performed alone, is not an effective treatment for OSA [11], while positional therapy is often difficult to sustain. On the other hand, he put on weight. Therefore, the worsening of sleep respiratory disorders was not unexpected. The onset of systemic hypertension and depression may have been a complication of his untreated OSA [12, 13]. Besides, a high percentage of REM sleep during CPAP titration could be foreseen, as REM rebound is more likely in patients with severe disease [3]. The surprising finding was the amount of the REM rebound. To our knowledge, so far a REM rebound due to CPAP application with REM sleep reaching 72% of the total sleep time has not been described, and we immediately realized that it could not have been caused by CPAP application alone. We enquired about possible additional factors contributing to that abnormal REM rebound and concluded that withdrawal of paroxetine could be involved.

In healthy adults REM sleep represents about 20% of the total sleep time. It recurs approximately every 90 minutes and progressively increases its duration in consecutive sleep cycles. Several causes increase the percentage of REM or of the REM density during the night: first application of CPAP in patients with OSA [2], some stressful situations [14], depression [15], and withdrawal of substances suppressing REM, like alcohol [16], cocaine [17], or antidepressive drugs [18]. Among antidepressive drugs, the selective inhibitors of serotonin uptake (SSRI) decrease slow wave sleep and REM sleep and prolong REM sleep latency [19]. SSRI withdrawal, especially if it is not performed gradually, is followed by a shortening of the REM sleep latency and an increase in total REM duration. In particular, paroxetine withdrawal is followed by a powerful REM rebound, which may last a few nights [20, 21].

## 4. Conclusions

REM rebound could be of clinical relevance in OSA patients who are at risk of hypoventilation during REM sleep when CPAP treatment is initiated. This case suggests that initiation of CPAP treatment and withdrawal of REM suppressing substances may have additional effects on sleep architecture. No dangerous consequences of the excessive REM duration were observed in this patient. However, a very long REM duration could be of clinical relevance in OSA patients who are at risk of hypoventilation during REM sleep when CPAP treatment is initiated and who could be exposed to prolonged hypoxemia and hypercapnia during the night if appropriate measures are not readily adopted.
